# Using mHealth to Predict Asthma Exacerbations in Children and Adolescents (Mobile Health for Kids With Asthma): Protocol for an Observational Study

**DOI:** 10.2196/70517

**Published:** 2025-09-11

**Authors:** Naphtal Nyirimanzi, Myriam Bransi, François-Pierre Counil, Olivier Drouin, Jocelyn Gravel, Anne Hicks, Cristina Longo, Theo J Moraes, Esli Osmanlliu, Dhenuka Radhakrishnan, Connie Yang, Teresa To, Bruce Wright, Sze Man Tse

**Affiliations:** 1 Department of Biomedical Sciences Faculty of Medicine Université de Montréal Montreal, QC Canada; 2 Centre mère-enfant Soleil Centre Hospitalier Universitaire de Québec Québec, QC Canada; 3 Faculty of Medicine Université de Laval Québec, QC Canada; 4 Department of Pediatrics Division of Respiratory Medicine Centre Hospitalier Universitaire de Sherbrooke Sherbrooke, QC Canada; 5 Faculté de Médecine et des Sciences de la Santé Université de Sherbrooke Sherbrooke, QC Canada; 6 Department of Pediatric Emergency Medicine Centre Hospitalier Universitaire Sainte-Justine Montreal, QC Canada; 7 Department of Pediatrics and Department of Social and Preventive Medicine Faculty of Medicine Université de Montréal Montreal, QC Canada; 8 Department of Pediatrics Faculty of Medicine Université de Montréal Montreal, QC Canada; 9 Department of Pediatrics Division of Pediatric Respiratory Medicine University of Alberta Edmonton, AB Canada; 10 Centre de Recherche Azrieli Centre Hospitalier Universitaire Sainte-Justine Montreal, QC Canada; 11 Faculty of Pharmacy Université de Montréal Montreal, QC Canada; 12 Division of Respiratory Medicine and Program in Translational Medicine Hospital for Sick Children Toronto, ON Canada; 13 Department of Pediatrics University of Toronto Toronto Canada; 14 Department of Pediatrics Division of Emergency Medicine McGill University Montreal, QC Canada; 15 Research Institute Children's Hospital of Eastern Ontario Ottawa, ON Canada; 16 Department of Pediatrics University of Ottawa Ottawa, ON Canada; 17 Child Health Evaluative Sciences Hospital for Sick Children Toronto, ON Canada; 18 Dalla Lana School of Public Health University of Toronto Toronto Canada; 19 Department of Pediatrics Division of Pediatric Emergency Medicine University of Alberta Edmonton, AB Canada; 20 Department of Pediatrics Division of Respiratory Medicine Centre Hospitalier Universitaire Sainte-Justine Montreal, QC Canada

**Keywords:** children, asthma, prediction, exacerbation, multimodal data

## Abstract

**Background:**

Asthma exacerbation is a major cause of emergency department visits in children and adolescents. Most of the existing asthma prediction scores and biomarkers are designed to predict severe exacerbations in the medium to long term. Mobile health (mHealth) is a promising approach for integrating real-time, multimodal data to improve the prediction of asthma exacerbation. Using mHealth can enable the identification of at-risk children and the implementation of timely interventions.

**Objective:**

The primary objective of the Mobile Health for Kids With Asthma (MoKA) study is to develop a validated predictive model for imminent asthma exacerbation in children using multimodal data, including participant-reported questionnaires through the RespiSentinel mobile app, augmented with publicly sourced environmental and epidemiological data. Furthermore, we will evaluate the association between the frequency of nocturnal cough measured in real time and asthma control and severe asthma exacerbation, and the acceptability of the RespiSentinel app in asthma self-management.

**Methods:**

This is a prospective cohort study with in-person and remote recruitment at 7 tertiary pediatric centers in Canada. Parents of children aged between 1 and 17 years, as well as children who have experienced at least one wheezing episode or asthma exacerbation during the 12 months before recruitment, will be eligible to participate (estimated number of children: n=2000). The planned duration of study participation is 6 months following the date of enrollment (cohort entry), regardless of the number of asthma exacerbations during the follow-up period. The primary outcome will be asthma exacerbation defined by asthma symptoms requiring systemic corticosteroid use and an urgent care or emergency department visit or hospitalization. The predictive model will be created using questionnaire data on asthma control via the RespiSentinel app as well as by integrating publicly available local daily data on air pollutant levels (National Air Pollution Surveillance Program) and weekly prevalence of respiratory viruses (National Canadian Respiratory Virus Detection Surveillance Program). Nocturnal cough frequency will be determined by using nighttime audio recordings, and their contribution to predict imminent asthma exacerbation will be evaluated. Acceptability of the RespiSentinel app will be assessed through an app-based questionnaire.

**Results:**

We will train and validate an asthma exacerbation prediction model using multimodal data sources. This approach may help patients, their families, and health professionals anticipate upcoming loss of asthma control and take the necessary steps to prevent a severe asthma exacerbation.

**Conclusions:**

The MoKA study will harness real-time mHealth data to identify children at imminent risk of asthma exacerbation with the ultimate goal of designing timely interventions to prevent morbidity in this group of patients.

## Introduction

### Background

Asthma is the most common chronic inflammatory respiratory disease in children and young adults, manifesting with cough, wheezing, and respiratory distress [1]. Globally, asthma affects more than 300 million people [2-4], including approximately 81 million children [5], with the prevalence expected to reach 400 million people by 2025 [2]. Severe asthma exacerbations, defined as a loss of baseline control requiring systemic corticosteroids, an emergency department (ED) visit, or a hospitalization [6], cause a significant burden to patients, their families, and the health system [4,7]. Exacerbations lead to school and parental work absenteeism, lower quality of life among children and their caregivers, and are a significant contributor to health care use and individual and societal costs [8,9]. Severe exacerbations are common, with approximately 10% of Canadian children with asthma having had 1 or more asthma-related ED visits in the previous 2 years [10,11]. Asthma morbidity and mortality are higher in children and adolescents compared with adults [12], and morbidity is particularly high in preschool-aged children [13,14] who represent >50% of all asthma-related ED visits [10].

### Risk Factors for Asthma Exacerbations

There are several known risk factors for severe asthma exacerbations. These can be grouped into clinical factors such as poor asthma control [15,16], previous exacerbations [17], and obesity [18] and patient-related factors such as poor medication adherence [19,20], socioeconomic status [21,22], comorbid allergic diseases [23,24], and genetic predispositions [25]. Furthermore, some environmental factors, including exposure to tobacco smoke [26,27] and indoor or outdoor allergens and pollutants [28,29], contribute to asthma exacerbations. Being able to predict severe asthma exacerbations in the short term could lead to timely interventions including improved adherence to controller medications, avoidance of certain exposures, and timely access to medical care. While various models have been developed to predict asthma exacerbations based on the risk factors and patient clinical profiles [30], existing pediatric predictive scores are limited by the integration of only a subset of these risk factors [15,17] and the lack of real-time data. Therefore, most studies aim to predict exacerbations over the following months or years. Other predictive models include objective measures such as oscillometry [31,32], spirometry and the fraction of exhaled nitric oxide [33-35], blood biomarker levels [36], and exhaled breath condensate analysis [37,38]. However, these measures are not easily accessible outside of pediatric centers or have only been used in a research setting. Oxygen saturation can be reliably and readily measured through mobile apps [39]; however, its use has been studied during acute asthma exacerbations and using hospital-grade devices [40,41]. Oxygen saturation, including its use in composite scores such as the Pediatric Respiratory Assessment Measure [42] and the Modified Pulmonary Index Score [43], is predictive of exacerbation severity and the risk of hospitalization. However, baseline oxygen saturation has not been studied as a predictor of asthma exacerbation. Thus, there is a need for simple and accessible predictive tools that can be applied to a representative sample of children with asthma in a timely fashion to create a significant impact on their care.

### Mobile Health and Asthma

Mobile health (mHealth), a subset of telehealth defined as the use of mobile devices for medical practice, is a promising approach that promotes the self-management of chronic conditions, including respiratory diseases [44,45]. mHealth can provide patients with unlimited access to health data and knowledge and provide physicians with remote monitoring of patients. Studies have shown that patients who use mHealth for various chronic respiratory conditions report greater self-awareness and self-confidence in disease monitoring [46] and reduced health care use, with improved health outcomes [47]. Parental perspectives on mHealth are positive [48,49], though few mobile tools are available to parents of children with chronic respiratory conditions. Physicians report better patient engagement and care outcomes with the use of mHealth compared with standard care [50,51]. As a research tool, mHealth can collect real-time and real-world patient-reported data, which can be used for disease phenotyping, to assess treatment response, and to predict adverse outcomes [52,53]. These data can be harnessed to identify children at high risk of impending asthma exacerbations.

While patient-reported data are valuable predictors of exacerbations, the addition of an objective measure can improve predictive models. Although lung function is a commonly assessed objective measure in children, it is challenging to measure in younger children, it has limited accessibility outside of clinic visits, and its utility in predicting future exacerbations in young children has not been shown [16,30,54]. Conversely, nocturnal cough is an easily measured and common parent-reported symptom in the prodrome period before asthma exacerbation, as cough frequency and intensity usually increase in the days leading to the exacerbation [55,56]. Studies have shown that the patterns of nocturnal cough are associated with asthma control and the detection of possible asthma exacerbation [56,57], with an increased occurrence of cough at the beginning of night sleep, particularly in the first 30 minutes to 1 hour [56,58]. With the accessibility of sound acquisition through mobile phone microphones and techniques in artificial intelligence allowing scalable and automated cough detection [59], home recordings of cough frequency [60-62] could be an additional objective tool that can be integrated into an mHealth app to predict asthma exacerbations, although more studies in children are required to validate its use.

To address these knowledge gaps, we will conduct the Mobile Health for Kids With Asthma (MoKA) study, an app-based prospective study to predict severe asthma exacerbations in children using multimodal data. This study will use the RespiSentinel mobile app, which our team created specifically for the MoKA study. RespiSentinel is an Android- and iOS-compatible mHealth app that allows the collection of patient-reported research data through the completion of questionnaires. While it contains useful and curated information on asthma, the RespiSentinel app allows automatic cough recordings and comprises several features including reminders on medication refill and intake, as well as the assessment of asthma control.

The MoKA study has three objectives: (1) to train and validate a model to accurately predict the risk of an imminent severe asthma exacerbation by integrating multimodal data (ie, self-reported asthma symptoms, comorbidities, medications and adherence, environmental data, and respiratory virus prevalence data); (2) to explore the association between nocturnal cough frequency evaluated through automated detection and asthma exacerbation within the following week in children and to integrate the cough patterns into the predictive model; and (3) to assess the acceptability of the RespiSentinel app among participants.

## Methods

### Study Design and Participants

The MoKA study is a multicentric, app-based prospective cohort study funded by the Canadian Institutes of Health Research (application 469005) with remote and in-person recruitment. We will use social media publicity campaigns to target eligible participants across Canada and will conduct in-person recruitment in respiratory and asthma clinics, EDs, or hospitalization units at 7 tertiary care pediatric centers in Canada: the Centre Hospitalier Universitaire Sainte-Justine (CHUSJ), Montreal; Montreal Children’s Hospital, Montreal; Centre Hospitalier Universitaire de Québec-Université Laval, Quebec; Centre Hospitalier Universitaire de Sherbrooke, Sherbrooke; the Children’s Hospital of Eastern Ontario, Ottawa; the Hospital for Sick Children, Toronto; and the Stollery Children’s Hospital, Edmonton. Inclusion criteria include (1) parents whose children are aged between 1 and 13 years and adolescents aged between 14 and 17 years; (2) children or adolescents with one or more wheezing episode or asthma-related ED visit or hospitalization in the past 12 months for which systemic or inhaled corticosteroids or a bronchodilator was prescribed; (3) living in Canada; and (4) understanding English or French. While the recruitment strategies may differ by site, in general, research assistants will identify potential participants based on eligibility criteria through electronic medical records. Then, they will contact the eligible participants, explain the purpose and procedure of the study, and invite them to participate in the study. These eligible participants may ask as many questions as they wish for further clarification before signing the voluntary e-consent form to enroll in the study. After downloading the RespiSentinel app, they will create an account linked to their phone number, as a validation code is sent to the participant’s phone at each log-in, for security purposes. The participant will be able to personalize the app by selecting optional notifications (eg, medication administration reminders) and choosing the day of the week they would like to receive the questionnaires. This personalization does not interfere with data collection for the MoKA study but offers the participants a choice in the use of nonstudy features.

Recruitment began in October 2023 with a pilot recruitment phase at CHUSJ and developed to full recruitment by January 2024 at CHUSJ. The integration of the other sites is ongoing. We anticipate recruitment to end in December 2026.

**Figure 1:**
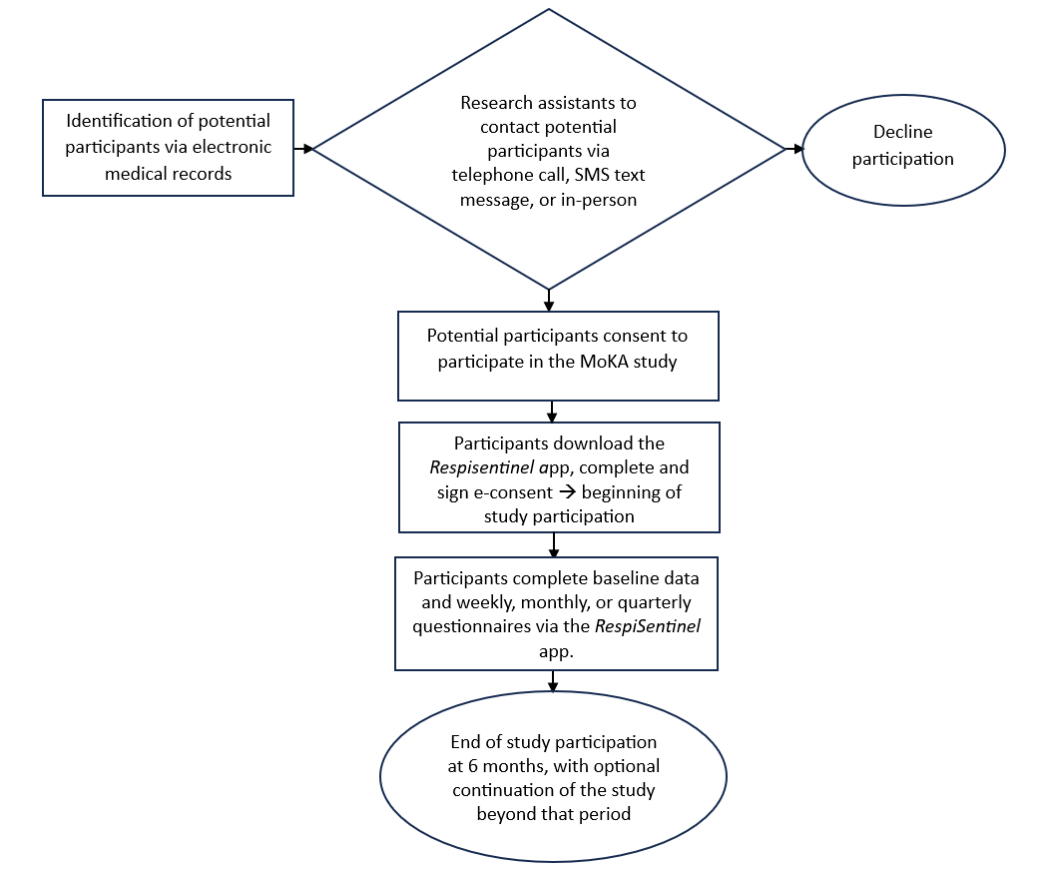
Mobile Health for Kids with Asthma (MoKA) study procedure.

For objective 2 on the automated cough detection, children sharing a room with another person will be excluded because of the complexity of sound analysis in that setting. Informed consent (and assent from adolescents, when appropriate) will be obtained from parents. In some participating institutions, this study is judged to be minimal risk and allows consent to be given by adolescents (usually aged ≥14 years) with asthma. In such cases, a copy of the consent form can be sent to the parents. Cohort entry (ie, start of follow-up) will be defined as the date on which children meet all inclusion criteria and informed consent is obtained. The participants are expected to remain in the study for 6 months after cohort entry and will have follow-ups throughout this period, regardless of whether they experience asthma exacerbations during the follow-up period (Figure 1). Furthermore, the participants will have the option to continue the study for an additional 6 months if they wish.

### The RespiSentinel App

**Figure 2:**
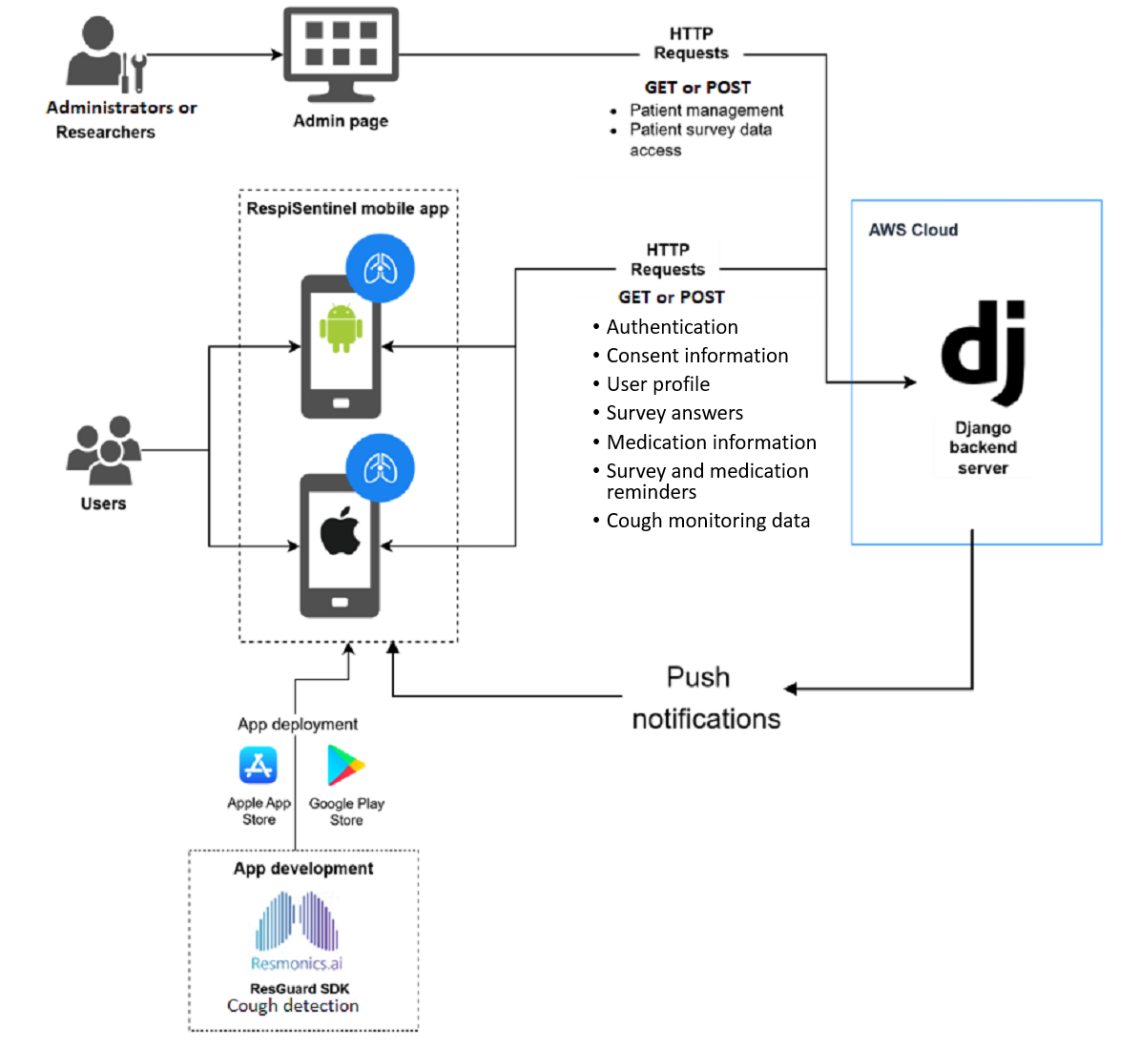
Simplified architectural presentation of the RespiSentinel app. Researchers and administrators of the RespiSentinel app manage participant data through an administrator page. Access is restricted using 2-factor authentication. Resmonics’ ResGuard software development kit is integrated into the RespiSentinel app, which is available on the Apple App Store and Google Play Store. The data entered by participants are stored on the Amazon Web Services (AWS) cloud. A Django backend server provides the framework for data management, including push notifications that are sent to participants as reminders to complete questionnaires.

Built specifically for the MoKA study and published by CHUSJ, RespiSentinel is a free, accessible Android- and iOS-compatible app that allows for the collection of patient-reported research data through the completion of questionnaires by parents of children with asthma or adolescents with asthma themselves. The overall architecture of RespiSentinel is provided in Figure 2. Following a short questionnaire screening for eligibility to participate in the MoKA study, an e-consent form approved by the research ethical board (REB) of CHUSJ is made available directly within the app. After consenting, a PDF version of the consent form is accessible through the app at any time (or can be sent to the participant’s email upon request). Once consented, the participants complete 2 baseline questionnaires, one on their general health and demographics and another on their asthma clinical history, current therapy, and environmental exposure. Subsequently, through in-app notifications sent to the participants at predetermined frequencies, they are prompted to complete weekly questionnaires on their general asthma symptoms and control, and monthly questionnaires on their use of asthma-related health resources, lung function, and overall health. The participants are prompted to answer a questionnaire every 3 months, asking for their feedback and comments on the RespiSentinel app. For the automated cough detection module, a licensed software development kit was provided by Resmonics [63], which was integrated into the RespiSentinel app by the app developers. Resmonics has developed a validated cough detection algorithm for adults [56,64], which is being used in ongoing studies for children [65].

In addition, several patient-oriented and desired features are available within the app, which were identified through a previous survey of 200 parents of children with asthma [66,67]. These features include curated educational videos on symptom recognition and inhaler use techniques. The RespiSentinel app includes the ability to track asthma symptoms and control, monitor medication intake, receive personalized alerts on medication renewals, and record significant events such as exacerbations and medical visits. The data on asthma control will be presented in a user-friendly graphical form that can be downloaded (eg, to share with their physician at a medical visit). Missing or erroneous data will be minimized through push notifications reminding participants to complete questionnaires via the app.

### Study Outcome Measures

The primary outcome for objectives 1 and 2 will be an imminent severe asthma exacerbation, defined as an increase in asthma symptoms leading to the use of systemic corticosteroids and an urgent care visit, ED visit, or hospitalization in the following week [68]. The occurrence of a severe exacerbation (ie, a dichotomous variable) will be assessed through a monthly parent- or self-reported questionnaire in the RespiSentinel app during the follow-up period*.* Specifically, this will be assessed through 3 separate questions: “In the past month, did your child go to an urgent care or walk in clinic for asthma?”, “In the past month, did your child go to the emergency department for asthma?” and “In the past month, was your child hospitalized for asthma?” If the participant answers “yes” to any of these 3 questions, the self-reported exact date of the medical consultation for the exacerbation and whether the child received oral or systemic corticosteroids for the asthma exacerbation will be collected. Given that participants are not necessarily followed for their asthma at the recruiting sites and can seek care at any point of care, self-reported exacerbations will be used, which have been shown to correlate well with administrative data [69], even over longer recall periods. A participant will be considered to have had the primary outcome of severe exacerbation if they had an asthma-related urgent care visit, ED visit, or hospitalization requiring corticosteroids. If a participant has an exacerbation during the follow-up period, we will instate a 2-week lag period following the date of the exacerbation during which the participant will not contribute to the prediction model. This will avoid counting an ongoing severe exacerbation as a new event, thereby preserving the model’s purpose of identifying and potentially intervening with children at high risk of an imminent exacerbation.

For objective 3, the outcome is the acceptability of the RespiSentinel app. The assessment of the acceptability of the RespiSentinel app by users will focus on the usability of the current version of the app (a posteriori acceptability) by assessing the depth, amount, breadth, and duration of user engagement through the frequency and number of measures recorded, the number of log-ins, the time spent on the app, and the number of pages accessed. We will also assess the a priori acceptability of the future version of the app, which will integrate an exacerbation risk score and automatic cough detection, after presenting the participants with a vignette describing these features. We will use a 5-point Likert scale and free-text comments to assess the a priori and a posteriori acceptability of the RespiSentinel app. The assessment will focus on various areas, including but not limited to the usefulness of the RespiSentinel app in tracking asthma symptoms and medications intake, the feedback on asthma control, the ability to share asthma control trends with clinicians, the ability to communicate the risk of asthma exacerbations, and the visual or graphic depiction of asthma control. A systematic review found an overall acceptability of mHealth apps in asthma of 3.32 (SD 0.64) using a 5-point scale [70]. A score of ≥3.5 (≥70%) will be considered acceptable.

### Independent Variables

For the prediction of severe asthma exacerbations, asthma control will be defined using an age-appropriate (age <6 or ≥6 years) 4-item Global Initiative for Asthma criteria for parent- or self-reported asthma control [1] and adapted for weekly assessments. The participants will respond “yes” or “no” to the 4 items focusing on whether the child had daytime symptoms, nighttime asthma symptoms, used asthma rescue medicines, or had a limitation of activities because of asthma during the previous week. The number of positive criteria will be used as a discrete variable and to categorize asthma as uncontrolled (3 or 4 positive answers), partly controlled (1 or 2 positive answers), or well controlled (no positive answers) [1]. In addition to asthma control, we will collect the following participant-reported variables: sociodemographic characteristics, comorbidities (eg, eczema and allergies), prematurity, the time from the last asthma exacerbation, tobacco and vaping exposures, asthma control (as defined earlier), and asthma therapy (ie, type and dose) and adherence to controller therapy through the number of weekly self-reported missed doses.

Furthermore, we will integrate local temperature, humidity, and aeroallergen levels when available, air quality indexes and air pollutants based on residential postal code, and weekly provincial prevalence of 7 common respiratory viruses. Specifically, the daily levels of nitrogen dioxide, ozone, sulfur dioxide, and fine particulate matter (PM2.5) from the monitoring station closest to the residential home will be considered. The provincial surveillance data on respiratory viruses include influenza viruses (A and B), respiratory syncytial virus, human rhinovirus or enterovirus, human parainfluenza virus, human metapneumovirus, human coronavirus, adenovirus, and SARS-CoV-2 [71]. This publicly available data will be obtained retrospectively through external sources such as Environment Canada [28], the National Air Pollution Surveillance Program [26], and the National Canadian Respiratory Virus Detections Surveillance Program [72], and retrospectively integrated into the database during the analyses.

Spirometry (ie, the forced expiratory volume in 1 second [FEV_1_], forced expiratory flow at 25%-75% [FEF_25-75_], and forced vital capacity [FVC]) and oscillometry (ie, resistance at 5 Hz) results from clinic visits will be integrated when available. To do so, participants will be asked to upload a picture of their test results in the app, if available. Research personnel will manually enter these data into the database. We acknowledge that many children will not have access to regular pulmonary function testing. This variable will be treated as optional, and a subgroup analysis will be performed among participants for whom these data are available.

Automated detection of nocturnal cough (ie, times per hour) through Resmonic’s software development kit for cough detection will be integrated into the RespiSentinel app [73]*.* This cough detection algorithm has been previously validated in adults but not yet in children. Parents will be invited to audio record the first 2 hours of sleep, in line with previous studies [56,58], weekly at a minimum for baseline reference and daily when the child is symptomatic (eg, at the start of a respiratory infection or symptoms caused by other triggers). The recording will be done through any mobile device equipped with a microphone that can be left at the bedside and on which the RespiSentinel app is loaded (eg, tablet or smartphone). While the participants must start the recording, no further interventions are required as the recording will automatically shut off after 2 hours and detect the cough episodes during that period. Parents or adolescents will fill out a questionnaire on the sleeping environment (ie, sleeping alone or with another individual) and the use of asthma control and reliever medication on the day of the recording.

### Study Procedure

Several recruitment strategies will be considered, based on the feasibility at each site. Figure 1 depicts the study procedure. First, we will advertise the study in pediatric respirology and asthma clinics and conduct a social media campaign. Second, we will approach patients presenting with asthma in the respiratory clinics, EDs, and hospitalization units at the participating centers. Third, eligible patients identified through their medical chart (eg, ED databases) will be contacted via SMS text messages inviting them to participate in the MoKA study. The participants will download the RespiSentinel app on a mobile device. e-Consent, based on best practices [74], will be provided to the participants through the app, with a copy sent to the participant. For adolescent participants, a copy of the consent form may also be sent to a parent. Once logged in using their mobile number and a unique code sent to the participant by SMS text message, the user can personalize their child’s profile (or their own, if an adolescent is answering for themselves) by providing information on asthma triggers, comorbidities, and current asthma therapy. Questionnaires will be pushed to the users via the app at a predetermined frequency. We will ask the parents to record their child’s cough through the app as previously described. The recording is programmed to shut off after 2 hours automatically. External data will be integrated into the database retrospectively.

### Data Analysis

The results will be reported using the STROBE (Strengthening the Reporting of Observational Studies in Epidemiology) [75] and CONSORT-EHEALTH (Consolidated Standards of Reporting Trials of Electronic and Mobile Health Applications and Online Telehealth) guidelines [76]. We will describe the baseline characteristics, retention, and analytic indicators of user engagement, including the number of measures recorded by users, frequency of interactions, number of features accessed by users, number of log-ins, and time spent using the app. Multiple imputation will be applied to manage missing data using random forest imputation for epidemiological data [77,78].

For objective 1, we will train and validate a predictive model for an imminent severe asthma exacerbation using an 80:20 approach (ie, 80% of the data for training and cross-validation and 20% for testing). We will standardize time-varying and fixed variables including self-reported asthma control, baseline comorbidities, exposures, asthma therapy, medication adherence, pulmonary function results, and external data, such as temperature, humidity, air quality indexes, and provincial virus prevalence data for inclusion in the prediction model. For time-varying variables, data from the week before asthma exacerbation will be considered. An extreme gradient boosting ensemble decision tree learning algorithm using binomial logistic regression will be trained and hyperparameters (ie, the number of trees, maximum tree depth, and the number of features considered at each split) optimized using grid search and 10-fold cross-validation within the 80% training split [79,80]. We will assess the overall model performance and the generalizability using the area under the receiver operating characteristic curve (primary measure), *F*_1_-score, precision, and recall from the 20% test dataset. The covariate importance will be assessed using a feature or covariate importance plot and Shapley additive explanations values [81,82]. In secondary analyses, we will also assess and compare the performance of other machine learning algorithms, such as support vector machines, random forests, naïve Bayes, and neural networks.

For objective 2, the integrated software will automatically classify events into cough and noncough events from raw audio data and automatically calculate cough frequency per hour [56,58]. We will estimate the association between cough frequency and asthma exacerbation in the forthcoming week using a generalized estimating equations approach to binomial logistic regression, adjusting a priori for other studied potential predictors of asthma exacerbations included in the predictive model. In secondary analyses, we will estimate the association between cough frequency and asthma control using a generalized estimating equations approach to negative binomial regression.

For objective 3, we will perform descriptive analyses of acceptability measures, including percentages for each category on the Likert scale and the participant-desired features. Using term frequency–inverse document frequency from the Python (Python Software Foundation) scikit-learn library [83], we will also extract key points from user comments to guide the development of the future app.

### Study Sample Size

On the basis of the previously published data from the CHUSJ [84] and data from the latest months of recruitment at CHUSJ, we estimate a general recruitment pool of approximately 5000 children with asthma-related ED visits per year and an 8% consent rate for the MoKA study. While recruitment methods and consequently general recruitment pools vary from one recruiting site to another, based on this consent rate and assuming a general recruitment pool of 10,000 patients per year across all sites, we expect to recruit a sample of 2000 participants (an average of 667 participants per year over a 3-year recruitment period). Extreme gradient boosting can detect an area under the receiver operating characteristic curve >90% with sample sizes that are smaller than this, including models with 20 to 110 predictors [79,85]. Therefore, we plan to recruit a sample of 2000 children with asthma over 3 years. On the basis of previous studies, severe exacerbations occur in up to 50% of preschool-aged children [13] and 25% of school-aged children [72] who had an exacerbation in the previous year. To enhance the generalizability of our findings, we aim to recruit approximately 70% (1400/2000) preschool-aged children and 30% (600/2000) school-aged children, with an estimated average of 30% (600/2000) of children developing an asthma exacerbation. Because the required sample size depends on the complexity of the algorithm for data analysis, we will also construct a learning curve a priori [86] (describing the classifier performance relative to training sample size) using the first 250 and 500 patients. This will help determine the required sample size and avoid additional recruitment and resource use once the classifier algorithm has reached its efficiency threshold. For objective 2, in adults, nocturnal cough frequency has a sensitivity of 75% and specificity of 70% for predicting asthma exacerbations 4 days later [56]. Assuming 30% of participants will experience an asthma exacerbation, we aim to recruit a minimum of 270 children with cough data.

### Ethical Considerations

The study has been approved by the CHUSJ REB (MP-21-2023-4333) and approval was sought from the REBs at participating centers before the start of recruitment at these sites. Although interested and eligible parents of children with asthma and adolescents can self-consent through the REB-approved e-consent form in the app, the research team will contact participants by telephone to ensure their understanding of the study and answer any questions that they may have. This approach will ensure informed consent and assent. Participants will have the option to withdraw from the study at any time if they wish. As part of the MoKA study, participants will consent to have their data kept in the RespiSentinel databank for potential future use and access by the same or other researchers, in compliance with national, provincial, and institutional confidentiality and data protection policies and principles. A data access committee has been created for requests to access data in the RespiSentinel databank.

Coded data from the app, including questionnaire answers and cough recordings, will be uploaded to the RespiSentinel databank on a secure server hosted by Amazon Web Services in availability zone 3, specifically located in the Central Canada region with Amazon CloudFront edge locations in Toronto, Ontario, and Montreal, Quebec. Data are stored via Amazon Simple Storage Service. Participants’ identifying information and the key linking this information to the coded data can only be accessed by members of the research team at the center that recruited them. To further ensure data confidentiality, different levels of data access will be restricted within the local team. The developers of the RespiSentinel app (third-party vendor bld.ai) do not have access to identifying information. The coordinating center (ie, CHUSJ) can only download coded data from all participating centers. To ensure data security, we have implemented additional protective strategies, such as data encryption during transfer and at rest, restricted access to the database via a Secure Shell key, and tracking of all activities in the database through CloudWatch. The data downloaded for analysis will be stored in password-protected files on a password-protected computer within a secure institutional server. There will not be any compensation to participate in this study, although monthly US $18 (equivalent to 25 Canadian dollars) gift certificates will be drawn among participants.

## Results

From October 1, 2023, to April 25, 2024, a total of 127 participants consented to the MoKA study, with a predominance of children aged <5 years (94/127, 76.4%), boys (82/127, 64.6%), and White participants (68/127, 53.5%). The mean age was 4 (SD 2.8) years. The baseline assessment revealed that 64% (35/55) of children had eczema, 7% (4/55) had rhinitis, and 20% (11/55) had food allergies. Atopic conditions were common among family members of participating children, with 68% (52/76), 38% (29/76), 35% (27/76), and 38% (29/76) of caregivers reporting asthma, eczema, allergic rhinitis, and environmental allergies, respectively. Among siblings, asthma and eczema were also common in 61% (19/31) and 48% (15/31), respectively. Regarding environmental exposures, 55% (29/53) of families had cats and 47% (25/53) had dogs. In addition, 14% (5/36) and 11% (4/36) of respondents reported cigarette or e-cigarette use, respectively.

In the 4 weeks before enrollment in the MoKA study, 70% (62/88) of participants perceived their child’s asthma as well controlled. However, based on specific questions on asthma control over the same period, 59% (52/88) and 36% (32/88) of participating children had daytime and nighttime asthma symptoms, respectively. Nearly half of the children (42/85, 49%) used salbutamol, and 34% (30/88) experienced limitations in activities because of asthma. In the 12 months before enrollment, 35% (31/88) of children had 1 asthma-related ED visit, 28% (25/88) had 2 asthma-related ED visits, and 18% (16/88) had ≥4 asthma-related ED visits. Asthma-related hospitalizations during the same period occurred in 35% (31/88) of children. Adherence to asthma control medications was 73% (64/88; defined as reporting daily or frequent use of their controller medication, or 5 to 6 days per week), with the most commonly used medications being fluticasone propionate and ciclesonide.

## Discussion

### Anticipated Findings

The MoKA study was designed to address two main knowledge gaps: (1) whether the use of nearly real-time data can improve the prediction of severe asthma exacerbations in children and (2) whether mHealth can be used to facilitate asthma management through the prediction of severe asthma exacerbations.

### Predicting Asthma Exacerbation

Several studies have reported predictive scores for asthma exacerbations in children, which integrated risk factors [15] and various objective measures [30,33,36]. However, the published models mostly evaluated longer-term predictions (eg, over the next year) using selected retrospective data, while our study aims to predict asthma exacerbations within the next week, thus allowing caregivers to act upon symptoms rapidly. Nonetheless, these prior studies provide insight into important risk factors, which we will collect and integrate into our model. A previous asthma exacerbation has consistently been identified as the most important predictor of future asthma exacerbations [87,88]. A systematic review of 26 studies found that previous asthma-related ED visits and hospitalizations were predictive of future asthma exacerbations [88]. Although the included studies were heterogeneous in defining exacerbation and the duration of follow-up, children with a history of exacerbation had up to 9.9 times the risk for a future exacerbation compared with those without a prior exacerbation [88]. Other studies have combined multiple risk factors in a single model to predict exacerbations. In a retrospective study using electronic medical record data collected from 3000 participants, Niu et al [15] built a risk score model to predict severe asthma exacerbations in the next 12 months in children and young adults. Allergic sensitization and exposure to smoke were major risk factors for preschool children, while obesity and abnormal spirometry were found to be predictive factors for asthma exacerbations in teenagers and young adults [15]. In another study using insurance claims data, Hatoun et al [89] derived an asthma exacerbation risk score to predict an asthma exacerbation in the next year. Their predictive model included age, presence of persistent asthma, prescription fill patterns of asthma medications and oral steroids, the number of outpatient visits, an exacerbation in the last 6 months, and whether spirometry was performed.

### Environmental Data in Prediction of Asthma Exacerbation

Environmental factors play an important role in triggering asthma exacerbations [90]. Thus, we have decided to include location-specific environmental data in our model. Most children with asthma present with allergic conditions, mainly to pollen and pets, and are sensitive to indoor and outdoor pollutants [91-93]. Several indoor exposures have been associated with the risk of asthma exacerbations in children [28,29], including tobacco smoke exposure, cockroaches, carpets, dust, and indicators of dampness. Various studies have demonstrated an association between outdoor air pollution levels, particularly PM2.5, ozone, and nitrogen dioxide and asthma exacerbations in children [94,95]. Interestingly, a study using a mobile app with connected devices conducted in 40 children found that exposure to higher levels of same-day PM2.5 and previous day ozone was associated with decreased lung function [96]. While the MoKA study will include these data retrospectively, the goal is to eventually include these variables in real time. Previous studies have identified important risk factors for asthma exacerbations, although they often evaluated a subset of these risk factors. In the MoKA study, we will integrate the aforementioned clinical, participant-reported, and environmental risk factors into our predictive model, which may optimize the prediction of upcoming asthma exacerbations and allow for timely interventions.

### Lung Function in Predicting Asthma Exacerbation

Other studies included objective measures such as lung function testing to predict asthma exacerbations in the medium to long term (ie, 3-12 months). Lung function, specifically low values of FEV_1_ and FEV_1_/FVC, and high airway resistance as measured by oscillometry, can predict future asthma exacerbations [31,33,97,98], although this association remains debated [99,100]. In addition, the fractional exhaled nitric oxide and bronchodilator response have predictive value for asthma exacerbations [33] and for loss of asthma control [34]. Other objective measures such as eosinophil counts as a marker of inflammation may also be useful in the prediction of exacerbations in children [36,101]. While lung function and biomarkers of inflammation may be useful in identifying children at risk for exacerbations, these measures are not easily accessible in children and are usually assessed at clinic visits, which does not allow the prediction of exacerbations in the short term.

### Innovations in Predicting Asthma Exacerbation

One novel aspect of the MoKA study is the exploration of automatic nocturnal cough detection as an objective measure in the prediction of poor asthma control and asthma exacerbations. Hirai et al [102] demonstrated that in children, exacerbations are characterized by increased cough frequency in the first 2 hours after falling asleep and at waking, with a median of 119 coughs per night. The cough count is also significantly higher in children with severe exacerbations compared with moderate exacerbations and compared with nonasthma controls [55]. However, these cross-sectional studies only examined cough during exacerbations without comparing it to intraindividual baselines. Sample sizes were limited as manual cough counting was labor intensive. The MoKA study will provide further insight into the usefulness of real-time automatic nocturnal cough detection through mHealth in predicting asthma exacerbations in children.

One strength of the RespiSentinel app used in the MoKA study is the availability of participant-centered and participant-desired resources, including curated educational materials and videos, and optional reminders to take and fill medications. These features were implemented following a survey of parents of children with asthma [66,67]. In addition to increasing participant engagement in the MoKA study, the initial feedback from participants was positive, with these resources helping them manage their child’s asthma. The acceptability of the RespiSentinel app as an mHealth tool will be formally assessed in the MoKA study.

### Limitations

Our study may incur a selection bias based on internet access. However, if a parent or patient declines participation solely because of their inability to access the internet, we will provide these participants with a refurbished phone and a pay-as-you-go internet access card. Thus far, in the MoKA pilot study and based on 2 previous mobile platform–based studies, we have not had any participants refuse participation based on this criterion. Self-selection bias or volunteer bias may also occur as participating families may be more inclined to engage with asthma management measures. As the possibility of selection bias is inevitable in any cohort study, even with traditional recruitment methods, this limitation will be acknowledged in our publications. Although the recall period is relatively short (1 week for asthma control and 1 month for asthma exacerbations), recall bias may nonetheless occur. To mitigate this risk, participants are encouraged to complete these questionnaires in a timely fashion through regular notifications. As with any self-reported data, social desirability bias may be an issue. To address this, participant anonymity and information confidentiality are ensured, the questionnaires were designed to be neutral, and participants are aware that data entered will not be shared with their treating physician. Self-report bias is possible as the participants will fill in information through the RespiSentinel app, particularly for perceived asthma control. This is mitigated by the evaluation of asthma control through a standardized symptoms-based questionnaire in addition to participant-perceived asthma control. We anticipate that there will be missing data and variable lengths of follow-up among the participants. To mitigate this, in addition to the reminders generated by the RespiSentinel app, we have set up a protocol to call or send email reminders to participants if they miss up to 3 consecutive questionnaires.

### Conclusions

The MoKA study harnesses mHealth to construct a model to predict asthma exacerbations in children and adolescents in a timely manner. Specifically, we built the RespiSentinel app to collect asthma-focused and patient-reported outcomes in real time. To facilitate participant engagement, the RespiSentinel app provides patient-oriented tools for asthma management that were developed with parents of children with asthma. Combined with externally sourced data and a novel automated cough detection tool, the predictive model derived by the MoKA study will offer the opportunity to inform users of their exacerbation risk in near real time using more comprehensive multimodal data. In addition, as an mHealth tool, the RespiSentinel app is a scalable infrastructure that integrates an e-consent form, push notifications to participants to answer questionnaires, data synthesis to provide summaries for the participants, and a data management platform. This infrastructure can easily be reused and adapted for other studies.

## Abbreviations

CHUSJ: Centre Hospitalier Universitaire Sainte-Justine
CONSORT-EHEALTH: Consolidated Standards of Reporting Trials of Electronic and Mobile Health Applications and Online Telehealth
ED: emergency department
FEV₁: forced expiratory volume in 1 second
FVC: forced vital capacity
mHealth: mobile health
MoKA: Mobile Health for Kids with Asthma
STROBE: Strengthening the Reporting of Observational Studies in Epidemiology
REB: research ethical board
